# Differential evolution-optimized gold nanorods for enhanced photothermal conversion

**DOI:** 10.1038/s41598-025-92007-7

**Published:** 2025-03-19

**Authors:** Aimad Koulali, Piotr Radomski, Paweł Ziółkowski, Francesca Petronella, Luciano De Sio, Dariusz Mikielewicz

**Affiliations:** 1 Faculty of Mechanical Engineering and Ship Technology, Institute of Energy, Gdańsk University of Technology, Narutowicza 11/12, 80-233 Gdańsk, Poland; 2 Institute of Crystallography CNR-IC, Montelibretti Division, National Research Council of Italy, Area Territoriale di Ricerca di Roma 1 Strada Provinciale 35d, n. 9, 00010 Montelibretti, RM Italy; 3 Department of Medico-Surgical Sciences and Biotechnologies, Sapienza University of Rome, Corso della Repubblica 79, 04100 Latina, Italy

**Keywords:** AuNRs, Optimization, DE algorithm, Photothermal conversion, CFD, Sustainable energy, Applied mathematics, Applied optics, Lasers, LEDs and light sources, Biomedical engineering, Mechanical engineering

## Abstract

Noble metallic nanoparticles (NPs) have shown great potential in the field of sustainable energy. Gold nanorods (AuNRs), known for their size-dependent optical and electrical characteristics, are strong candidates for various applications, particularly in solar energy conversion. Additionally, AuNRs are well-established nanomaterials in precision medicine. In this paper, we optimize the shape and size of AuNRs to maximize light-to-heat conversion based on a validated theoretical model. Utilizing the Differential Evolution (DE) algorithm, a robust metaheuristic optimization approach, we calculated the optimal size and shape of AuNRs for selected wavelengths. The aspect ratio (AR), defined as the ratio of the diameter to the length of the AuNRs, was a key parameter in the optimization process. The optimization results reveal that for shorter wavelengths, near-spherical AuNRs (AR of 0.71 and 0.75) demonstrate the highest efficiency, while for longer wavelengths, more elongated AuNRs (AR of 0.24 and 0.17) outperform others. This study also includes Computational Fluid Dynamics (CFD) calculations to evaluate the impact of optimized AuNRs on heat generation in a real-world scenario. A case study is presented in which lasers of different wavelengths irradiate a borosilicate glass embedded with a slab of AuNRs at its center. The results, reported as temperature distributions and temperature evolution during irradiation, indicate that the optimized AuNRs significantly enhance heat generation across various laser wavelengths. Specifically, temperature increases were observed as follows: from 2.28 to $39.08\,^\circ \textrm{C}$ at 465 nm, from 1.91 to $81.42\,^\circ \textrm{C}$ at 532 nm, from 1.7 to $65.14\,^\circ \textrm{C}$ at 640 nm, from 40 to $48.35\,^\circ \textrm{C}$ at 808 nm, and from 0.94 to $118.45\,^\circ \textrm{C}$ at 980 nm, respectively. These findings underscore the effectiveness of the optimization process in enhancing photothermal conversion.

## Introduction

A sustainable energy supply, including renewable sources such as solar energy, wind power, and bioenergy, is seen as one of the most important areas of concern for our planet at present. As declared by the International Energy Agency, 2020^1^, the transition to sustainable energy is critical in order to address global issues like climate change, resource depletion, and environmental degradation. Among the different approaches for sustainable energy, solar-induced energy has great interest based on its potential role in diverse industries. The principal driver of solar energy use is the efficient conversion of this energy into heat through photothermal processes. Therefore, research in this area is promising as it offers a way to develop innovative approaches and solutions for better light conversion into heat, which would greatly benefit environmental remediation. The phenomenon of the light conversion into heat is named the “photothermal effect”(PTE) for nanostructures. It refers to the preferential thermal relaxation over emissive relaxation of any quantum system that absorbs light^2^. The exceptional ability of nanomaterials to enhance the PTE has garnered significant attention, especially plasmonic nanoparticles (NPs). NPs absorb light much more intensely than molecules at specific resonances. In the case of noble metals, this characteristic is especially pronounced due to their ability to exhibit localized surface plasmon resonance (LSPR) at specific wavelengths^3^. The light absorbed at a maximum rate due to the LSPR is then converted into heat by the photothermal effect^4^. 

Photothermal conversion has several important biomedical applications. Photothermal therapy (PTT) is used for cancer treatment, where gold nanoparticles (AuNPs) target and destroy cancer cells by converting light into heat^5–10^. This technique necrotizes tumor cells without damaging surrounding healthy tissue. Plasmonic nanomaterials (e.g., AuNPs or AgNPs), are also used against antibiotic-resistant bacteria under light irradiation^11–15^. In addition, these nanomaterials can accelerate wound healing by improving blood circulation and stimulating tissue regeneration^16–19^. 

In addition, the heat generated by photothermal nanomaterials can be used to break down post-surgical adhesions, reducing pain and improving mobility after surgical procedures^20–24^. As mentioned by^25^, the vast majority of applied research has focused solely on cancer therapy and biomedical applications. In contrast, NPs can also play a key role in sustainable energy. Therefore, in the present work, we undertake a theoretical and fundamental investigation into optimizing the shape and size of one of the most widely studied NPs, namely AuNRs. This optimization aims to maximize heat generation, which can then be utilized across a broad spectrum of applications. In a review article^26^ on the photothermal conversion of solar energy using nanomaterials and nanostructures, the authors concluded that the photothermal conversion of solar energy is highly efficient, often exceeding 50%, making it a promising method for applications in thermal catalysis, water evaporation, desalination, bacterial destruction, and thermal response sensors. Other scientific reviews have also focused on using photothermal conversions in co-catalytic CO$_2$ reduction^27–31^, solar interfacial evaporation^32–35^, and anti-icing technologies^36–38^. 

Bearing in mind the above, it’s crucial to note that optimizing the photothermal conversion phenomenon is currently one of the main research objectives in the nanomaterial field^39^. By increasing the light absorption efficiency of NPs, this contributes to more efficient heat generation, and therefore more reliable applications^40^. Optimizing this phenomenon can be achieved through a variety of parameters, including the source of radiation (laser or white light), the concentration of NPs, or the shape and dimensions of the NPs irradiated, and eventually the surface coating. It is essential to note that a lot of effort has been invested in this research area to improve the outcome of the photothermal conversion phenomenon. The review of the literature shows that AuNPs have attracted particular attention, as these NPs have unique optical properties^41–44^. Liu and coworkers^45^ demonstrated that excellent optical properties of NPs are strongly related to their size and shape, making it crucial to select NPs based on their diffusion and absorption mechanisms to maximize PT performance in the target system. This relationship was further explored by Guo and colleagues^46^, who found that as the diameter of AuNPs increased from 3 to 40 nm, the photothermal conversion efficiency significantly improved, demonstrating a direct correlation between NPs size and the effectiveness of solar energy conversion for applications such as solar steam generation. Additionally, experimental studies conducted by Moustaoui and coworkers^47^ demonstrate that the absorption cross-section, which directly impacts the PTE, is highly dependent on the AuNPs’ surface area. For example, larger gold nanourchins (50, 80, and 90 nm) showed higher absorption, which allowed for more efficient local heat generation compared to smaller NPs. In the same work, the authors stated that the efficiency of nanosphere (AuNSs) in converting light into heat aligns well with theoretical predictions, confirming that larger surface areas enhance the PTE. 

AuNRs, known for their elongated structure, have shown remarkable efficiency in converting light into heat^48–50^, particularly when exposed to wavelengths within the near-infrared region (NIR), specifically between 750 and 950 nm^51^. This makes them highly effective in medical and photothermal applications. In contrast, AuNRs also exhibit stronger ultraviolet (UV) extinction below 220 nm, with properties that depend on the alignment of the light’s polarization with the AuNRs’ structure, whereas AuNSs typically display more uniform and size-dependent extinction across the UV range of 200 to 300 nm^52^. A study by Megan and colleagues^53^ found that among various sizes of AuNRs, the 28 $\times$ 8 nm size was particularly effective at generating heat when exposed to light. This size struck a good balance between absorbing light and converting it into heat, with an electric field that extends optimally from the particle’s surface, allowing for efficient heating through interactions between neighboring particles in solution.

 Further research, such as the study conducted by Henglei and colleagues^54^, has shown that even smaller AuNRs, with diameters less than 10 nm, can be highly effective for photothermal therapy (PTT). These small AuNRs, with inverse ARs (length/diameter) ranging from 2.7 to 4.7, exhibit a strong absorption-dominant behavior, with minimal contribution from light scattering (only 0.005 to 0.025 of the total extinction). The longitudinal plasmon resonance wavelengths of these AuNRs can be tuned between approximately 720 nm and 830 nm. Moreover, when coated with a dense silica layer and tested on three cell lines, these small AuNRs demonstrated superior photothermal therapy performance compared to larger AuNRs, indicating their potential as highly effective light absorbers and photothermal agents in biomedical applications. Beside, the study conducted by Varun and co-authors^55^ reveals that while gold nanoshells generate more heat per NP due to their larger size, AuNRs exhibit higher photothermal efficiency, making them more effective under certain conditions. 

However, there are some cases where AuNRs do not deliver the expected results. This was clearly demonstrated by Qin and coworkers^56^, who conducted a comparative study on the photothermal heat generation between AuNSs and AuNRs. They found that although AuNRs theoretically exhibit superior photothermal properties, the heat generation observed in their experiments was significantly lower than expected, primarily due to the effects of polydispersity. This finding underscores the importance of carefully considering the practical limitations of AuNRs, such as variations in size and shape. Similarly, in another study by Plowman and coworkers^57^, it was revealed that under certain conditions, AuNRs may exhibit less favorable electrochemical properties compared to NSs, particularly due to their tendency toward electrodissolution. Their findings suggest that while AuNRs may offer advantages in certain areas (e.g., plasmonic applications), their electrochemical behavior can be less stable or predictable than that of NSs.

**Figure 1:**
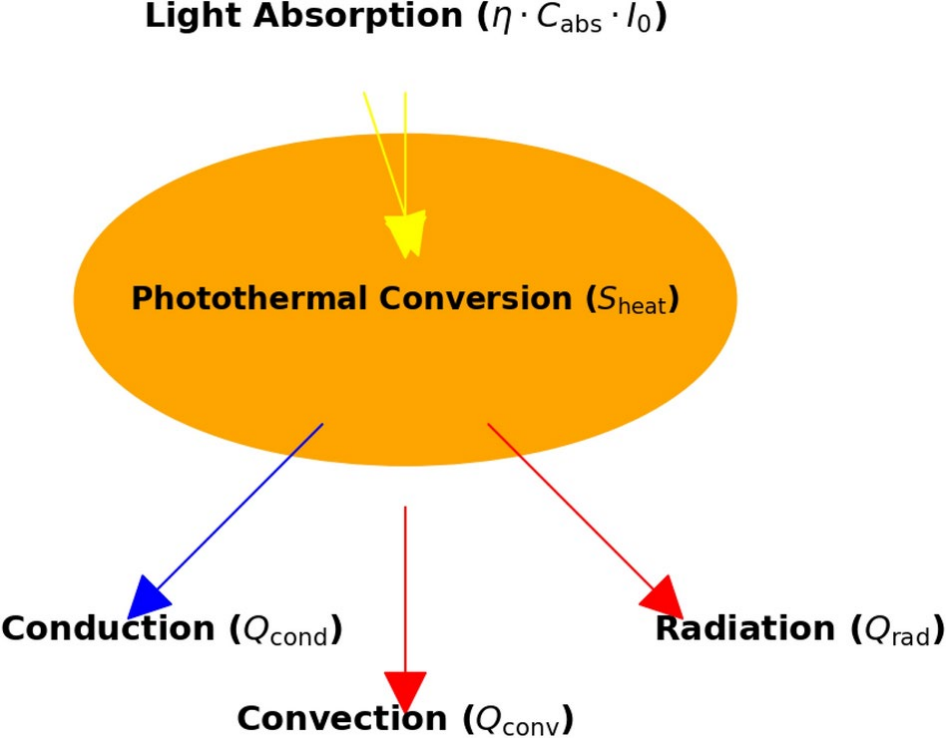
Simplified scheme of photothermal conversion.

To overcome the limitations of experimental studies that may occur during analysis of the photothermal efficiency of NPs, theoretical studies become essential. One of the most fundamental aspects to be considered when describing the photothermal conversion mathematically is the nature of light and its interaction with materials. In physics, light is considered as an electromagnetic wave, which can be characterized by its wavelength ($\lambda$), frequency (*f*) and energy (*E*)^58^. These parameters are correlated by the equation $E = h \cdot f$, where *h* is Planck’s constant^59^. Here, as light interacts with a material, part of the electromagnetic energy will be absorbed by the material, another part will be reflected and the rest can be transmitted through the material (see Fig. 1). The absorbed part of the electromagnetic wave in plasmonic NPs such as AuNPs excites surface plasmons, which are coherent oscillations of electrons at the surface. This excitation is the primary mechanism for converting light into heat. The photothermal conversion efficiency which quantifies the fraction of light energy that is converted into heat, can be correlated with the fundamental equation of heat generation in the photothermal process, and can generally be expressed as follows:

1 $$\begin{aligned} S_{\text {heat}} = A_{\text {abs}} \cdot \eta _{\text {TOT}}\cdot I_0 \end{aligned}$$

In Eq. (1), $S_{\text {heat}}$ is the heat generation rate, $\eta _{\text {TOT}}$ stands for the total conversion efficiency, $A_{\text {abs}}$ represents the absorption coefficient of the NPs, and $I_0$ is the incident light intensity. The most important parameter to be determined in this equation is the absorption coefficient $A_{\text {abs}}$, which quantifies the portion of incident light absorbed by the NPs. Several models and approximations have been proposed in the scientific literature to calculate $A_{\text {abs}}$. A common feature of these models is the consideration of the interaction between light and NPs, based on classical thermodynamics^60^.

In this paper, we apply the DE algorithm to optimize the shape and size of AuNRs when irradiated by different laser wavelengths, aiming to maximize heat generation. Our calculations are grounded in a validated model^15,61,62^, which has been cross-verified with experimental data through a combination of theoretical calculations and CFD simulations.

The first part of the paper outlines the theoretical model used to describe the conversion of light to heat in both the fluid medium and the AuNRs. Since this model has already been detailed in the authors’ previous work^61^, we only provide the essential equations and explanations, along with considerations for the role of size distribution in AuNRs.

The second part of the paper focuses on the model validation. This validation was carried out through two tests: first, by comparing the absorption characteristics of AuNRs as predicted by theoretical calculations against experimental data; and second, by comparing the temperature evolution over time when AuNRs are irradiated by a laser within a microfluidic chamber.

The third part of the paper presents the DE algorithm, explaining its nature as a metaheuristic approach and demonstrating its applicability to our model. We then present the optimization results, along with an assessment based on experimental AuNR samples that have been optimized using our findings.

The final part of the paper is dedicated to a real-world case study. In this study, we compare the experimental and optimized sizes of AuNRs by analyzing the temperature evolution in a borosilicate glass surface embedded with a layer of AuNRs, which is irradiated by lasers of different wavelengths.

This work represents a significant contribution to the field of photothermal conversion within the broader energy domain. It is the first time that an optimization process using theoretical calculations has been applied to this problem. Previous studies on the effect of NRs shape and size have primarily relied on experimental methods, which do not provide the exact size values needed for optimization across different wavelengths.

## Conversion of light to heat

### Light into heat conversion model

Nanomaterials’ conversion of light into heat, or photothermal conversion, can effectively raise their surrounding environment’s temperature in a spatially localized, non-contact way^63^. A wide range of nanomaterials can be explored in different applications, including organic and inorganic systems. Typical materials in the latter category are plasmonic NPs (e.g. AuNPs, AuNRs^64^). As pointed out by the authors^65^ in their paper on *Next-generation thermo-plasmonic technologies and plasmonic NPs in optoelectronics*, Au and Ag are the most promising nanostructures for optical and sensing applications. During the photothermal conversion process driven by gold NPs, light interacting with the gold NPs is scattered and absorbed, generating surface plasmon excitation. The spectral nature of the resulting absorbed radiation depends on numerous parameters, such as the shape, size, composition and environment of the NPs, and concentration^66^.

In addition, in the case of anisotropic NPs such as AuNRs, the angle of incidence and polarization of the light have a significant impact on the optical properties of non-spherical NPs. As long as the reflection coefficients can be described using the full Fresnel’s equations, absorption and scattering coefficient are modified due to anisotropy. For example, the plasmonic resonance of AuNRs depends on the alignment of the incident light’s electric field with the nanorod’s longitudinal and transverse axes. When the electric field is aligned with the longitudinal axis, longitudinal plasmon resonance dominates, leading to enhanced absorption and scattering. Conversely, alignment with the transverse axis results in weaker resonance. This sensitivity to angle and polarization can influence the heat generation efficiency and should be considered when designing applications involving anisotropic nanoparticles. Much more complicated problem appears when the surface effect is considered, and the surface is charged itself. At that case, the electric field distribution of nanostructures and their alignment to each other strongly affect the absorption and scattering cross section. Some of the results were touched in Zaccagnini’s research^15^. In this work, light energy converted into heat energy has been determined both in the fluid medium and also at the AuNRs. For the fluid part, the heat generation rate can be deduced based on the semi-infinite method and the Lambert-Beer-Bourger law^61,64,67,68^, the reader is invited to refer to our previous article^61^ where we have explicitly outlined the subject.

#### Fluid part

Taking Eq. (1) as the starting point, we can derive the equation for heat generation in the fluid medium under laser irradiation. First, the light absorption in the fluid should be adjusted, accounting for the reflection at the fluid interface^69^.

2 $$\begin{aligned} S_{\text {Ext/Tr, f}} = I_{0} \cdot (1-R_{\text {f}}) \end{aligned}$$

Here, $S_{\text {Ext/Tr, f}}$ represents the transmitted or extinguished signal through the fluid medium, $I_{0}$ is the incident light intensity, and $R_{\text {f}}$ refers the reflection coefficient of the fluid. Furthermore, the incident light intensity $I_0$ is given by:

3 $$\begin{aligned} I_0 = \frac{P_0}{\pi \cdot d_B^2} \end{aligned}$$

where:

- $P_0$: Power of the incident laser light.
- $d_B$: Diameter of the laser beam.

As stated by the Lambert-Beer-Bourger law^61,67,70^, the intensity of light decreases exponentially with the depth of penetration $l_{\text {s,f}}$ in an absorbing medium,

4 $$\begin{aligned} I(z) = I_0 \cdot \exp (-A_{\text {abs}} \cdot z) \end{aligned}$$

where:

- $I(z)$: Light intensity at depth $z$.
- $$A_{\text {abs}}$$: Absorption coefficient.
- $z$: Depth in the medium.

So, the absorbed depth in our case can be calculated as follows:

5 $$\begin{aligned} S_{\text {absorbed depth}} = S_{\text {Ext/Tr, f}} \cdot (1-\exp (-A_{\text {abs,f}} \cdot l_{\text {s,f}})) \end{aligned}$$

Using the previous Eqs. (2) and (5), the total heat generation in the fluid medium irradiated by a laser can be estimated by the following equation:

6 $$\begin{aligned} H_{\text {f}} = A_{\text {abs.f}} \cdot I_0 \cdot (1 - R_{\text {f}}) \cdot (1 - \exp (-A_{\text {abs.f}} \cdot l_{\text {s,f}})) \end{aligned}$$

#### AuNRs part

The heat generated by a single nanoparticle can be estimated using some basic concepts, starting from the fundamental equation for heat generation (Eq. 1). The generated heat model was already presented and physically well explained in the authors’ previous article^61^. Here, we present some essential concepts to help the reader understand the basic fundamental aspects of the model. Using the same Eq. 3 to define the incident light intensity in the AuNRs and Eq. 4 to describe the intensity of light as it decreases while traveling through an absorbing AuNR and taking into account the incident intensity adjusted by all reflection losses:

7 $$\begin{aligned} S_{\text {Ext/Tr, Au}} = I_0 \cdot \text {Refl}_{\text {outer}} \cdot (1 - R_{\text {wc}}) \cdot (1 - R_{\text {Au}}) \end{aligned}$$

Where, $\text {Refl}_{\text {outer}}$ is the reflection at the interfaces, $R_{\text {wc}}$ is the reflection coefficient at the water-core interface, and $R_{\text {Au}}$ is the reflection coefficient at the AuNRs surface^61^.

The absorption cross-section for AuNPs is given by^61,67^:

8 $$\begin{aligned} C_\text {abs,Au} = C_\text {ext,Au} - C_\text {sca,Au} \end{aligned}$$

Where, the extinction cross-section $C_{\text {ext,Au}}$ is related to the polarizability of AuNPs^61,67^:

9 $$\begin{aligned} C_{\text {ext,Au}} = 4 \pi \left( \frac{2 \pi }{\lambda }\right) \cdot \text {Im}(\alpha _{\text {shAu}}) \end{aligned}$$

and the scattering cross-section is given by^61,67^:

10 $$\begin{aligned} C_{\text {sca,Au}} = \frac{8}{3} \pi \left( \frac{2 \pi }{\lambda }\right) ^4 \cdot |\alpha _{\text {shAu}}|^2 \end{aligned}$$

The absorption cross-section $C_{\text {abs}}$ was first calculated by Mie^71–74^, who solved Maxwell’s equations to describe the scattering and absorption of electromagnetic waves by spherical particles. This parameter, which is central to the photothermal process, provides a direct measure of the particle’s ability to convert light energy into heat. For NPs, Mie theory is one of the most commonly used approaches for calculating $C_{\text {abs}}$. In addition to Mie’s theory, it is also possible to apply the Rayleigh approximation^75–78^, a simplified model of Mie’s theory, in the case of particles which are much smaller than the wavelength of light ($d_s \ll \lambda$). Therefore, Drude’s model^79^, which describes the optical and electrical properties of metals, can be combined with Mie’s theory to define the absorption properties of metal NPs, especially in the visible and NIR ranges. Likewise, the absorption cross-section could be determined from other models, such as the discrete dipole approximation^80^ and the finite-difference time-domain method^81^. The optical coefficients can be defined using the previous optical cross-section together with the concentration of AuNRs ($\xi _{\text {max}}$)^61^:

11 $$\begin{aligned} \xi _{\text {max}}(d_s,d_l) = \frac{2}{(d_s + \phi + 2d_{CT})^2 \cdot (d_l + \phi + 2d_{CT})} \end{aligned}$$

where, $\phi$ is the distance between NPs, $d_s$ with $d_l$ represent AuNRs diameter with length and $d_{CT}$ gives the shell thickness (here shell is capping agent). Then, the extinction, scattering, and absorption coefficients, are given as follows:

12 $$\begin{aligned} & \textit{A}_{\text {ext,Au}} = C_{\text {ext, Au}} \cdot \xi _{\text {max}} \end{aligned}$$

13 $$\begin{aligned} & \textit{A}_{\text {sca,Au}} = C_{\text {sca, Au}} \cdot \xi _{\text {max}} \end{aligned}$$

14 $$\begin{aligned} & \textit{A}_\text {abs,Au} = \textit{A}_\text {ext,Au} - \textit{A}_\text {sca,Au} \end{aligned}$$

The overall polarizability $\alpha _{\text {shAu}}$ of the AuNPs, including its shell, is defined as a combination of the polarizabilities along the *x* - axis and the *yz* -axes, weighted by the occupancy factor *OF*, where *x* - axis refers to the elongated dimension of NRs. For further information the reader can refer to^61,67^:

15 $$\begin{aligned} & \alpha _x = V(d_s, d_l) \cdot \nonumber \\ & \quad \frac{\left( n_{\text {sh}}^2 - n_{m1}^2\right) \left( n_{\text {sh}}^2 + \left( \tilde{n}_{\text {Au}} - n_{\text {sh}}^2\right) \cdot LL_x(d_s, d_l)\right) + f(d_s, d_l) \cdot \left( \tilde{n}_{\text {Au}} - n_{\text {sh}}^2\right) \cdot n_{\text {sh}}^2}{\left( n_{\text {sh}}^2 + \left( \tilde{n}_{\text {Au}} - n_{\text {sh}}^2\right) \cdot LL_x(d_s, d_l)\right) \cdot \left( n_{m1}^2 + \left( n_{\text {sh}}^2 - n_{m1}^2\right) \cdot L_x(d_s, d_l)\right) + f(d_s, d_l) \cdot L_x(d_s, d_l) \cdot \left( \tilde{n}_{\text {Au}} - n_{m1}^2\right) \cdot n_{\text {sh}}^2} \end{aligned}$$

16 $$\begin{aligned} & \alpha _{yz} = V(d_s, d_l) \cdot \nonumber \\ & \quad \frac{\left( n_{\text {sh}}^2 - n_{m1}^2\right) \left( n_{\text {sh}}^2 + \left( \tilde{n}_{\text {Au}} - n_{\text {sh}}^2\right) \cdot LL_{yz}(d_s, d_l)\right) + f(d_s, d_l) \cdot \left( \tilde{n}_{\text {Au}} - n_{\text {sh}}^2\right) \cdot n_{\text {sh}}^2}{\left( n_{\text {sh}}^2 + \left( \tilde{n}_{\text {Au}} - n_{\text {sh}}^2\right) \cdot LL_{yz}(d_s, d_l)\right) \cdot \left( n_{m1}^2 + \left( n_{\text {sh}}^2 - n_{m1}^2\right) \cdot L_{yz}(d_s, d_l)\right) + f(d_s, d_l) \cdot L_{yz}(d_s, d_l) \cdot \left( \tilde{n}_{\text {Au}} - n_{m1}^2\right) \cdot n_{\text {sh}}^2} \end{aligned}$$

17 $$\begin{aligned} & \alpha _{\text {shAu}}(d_s, d_l) = \textit{O}F\ \cdot \alpha _x(d_s, d_l) + (1 - \textit{O}F\ ) \cdot \alpha _{yz}(d_s, d_l) \end{aligned}$$

The parameters used in the equations above depend on the AuNRs diameter ($d_s$) and length ($d_l$) as follows (see Table 1 for more details):

- $V(d_s, d_l)$: Volume of the nanoparticle, including its shell (capping agent).
- $L_x(d_s, d_l)$ and $L_{yz}(d_s, d_l)$: Depolarization factors along the *x* and *yz* axes, respectively.
- $LL_x(d_s, d_l)$ and $LL_{yz}(d_s, d_l)$: Modified depolarization factors along the *x* and *yz* axes, respectively.
- $f(d_s, d_l)$: Volume fraction of the core in the total nanoparticle (core + shell).
- $n_{\text {sh}}$: Refractive index of the shell material (capping agent).
- $n_{m1}$: Refractive index of the surrounding medium (here: water ($n_{\text {Waterref}}$) or air ($n_{\text {Airref}}$, respectively).
- $\tilde{n}_{\text {Au}}$: Complex refractive index of gold, given by $\tilde{n}_{\text {Au}} = n_{\text {Au}} + i \cdot k_{\text {Au}}$.
- $\lambda$: Wavelength of the incident light.
- $OF$: Occupancy factor representing the orientation distribution of the AuNRs.

As it was stated by Zhang and Wang^82^, the optical properties of metal nanoparticle are strongly affected by factors likes nanoparticle size, shape, environment and concentration. Therefore, the need to adjust the optical coefficients defined by Eqs. (12), (13), and (14) to take into account the concentration of NPs and the saturation effect^61,67^. The adjusted optical coefficients are given by:

18 $$\begin{aligned} & \textit{A}_{\text {ext,Au,adj}} = \textit{A}_{\text {ext,Au}} \cdot \left( \frac{1}{1 + \frac{P_{0}}{P_{\text {sat}}}}\right) \end{aligned}$$

19 $$\begin{aligned} & \textit{A}_{\text {sca,Au,adj}} = \textit{A}_{\text {sca,Au}} \cdot \left( \frac{1}{1 + \frac{P_{0}}{P_{\text {sat}}}}\right) \end{aligned}$$

Moreover, the saturation power is experimentally given by^61^:

20 $$\begin{aligned} P_{\text {sat}} = 1.13 \cdot 10^{-23} \cdot \xi _{\text {max}} + 1.56 \end{aligned}$$

Therefore, the adjusted absorption coefficient can be formulated as follows:

21 $$\begin{aligned} \textit{A}_\text {abs,Au,adj} = \textit{A}_\text {ext,Au,adj} - \textit{A}_\text {sca,Au,adj} \end{aligned}$$

Attenuation of light within the AuNRs and shell, can be effectively presented using Lambert-Beer-Bourger law^61,70^:

22 $$\begin{aligned} S_{\text {absorbed depth,Au}} = S_{\text {Ext/Tr, Au}} \cdot (1-\exp (-\textit{A}_{\text {abs,adj,Au}} \cdot l_{\text {sh,Au}}\cdot \eta _{\text {PT}})) \end{aligned}$$

Here, $l_{\text {sh,Au}}$ is the thickness of the nanoparticle shell, is given by :

23 $$\begin{aligned} l_{\text {shAu}} = d_s + 2d_{CT} \end{aligned}$$

The photothermal efficiency $\eta _{\text {PT}}$ can be determined by:

24 $$\begin{aligned} \eta _{\text {PT}} = \frac{\textit{A}_{\text {abs,adj,Au}}}{\textit{A}_{\text {ext,adj,Au}}} \end{aligned}$$

Combining the equations above, the final heat generation equation for AuNRs is:

25 $$\begin{aligned} \begin{aligned} S_{\text {e}}(d_s,d_l) =&\, \textit{A}_\text {abs,adj,Au}(d_s,d_l) \cdot I_0 \cdot \text {Refl}_{\text {outer}} \cdot (1 - R_{\text {wc}}) \cdot (1 - R_{\text {Au}}) \\&\cdot \left( 1 - \exp \left( -l_{\text {shAu}}(d_s,d_l) \cdot \textit{A}_\text {abs,Au,adj}(d_s,d_l) \cdot \eta _{\text {PT}}(d_s,d_l)\right) \right) \end{aligned} \end{aligned}$$

**Figure 2:**
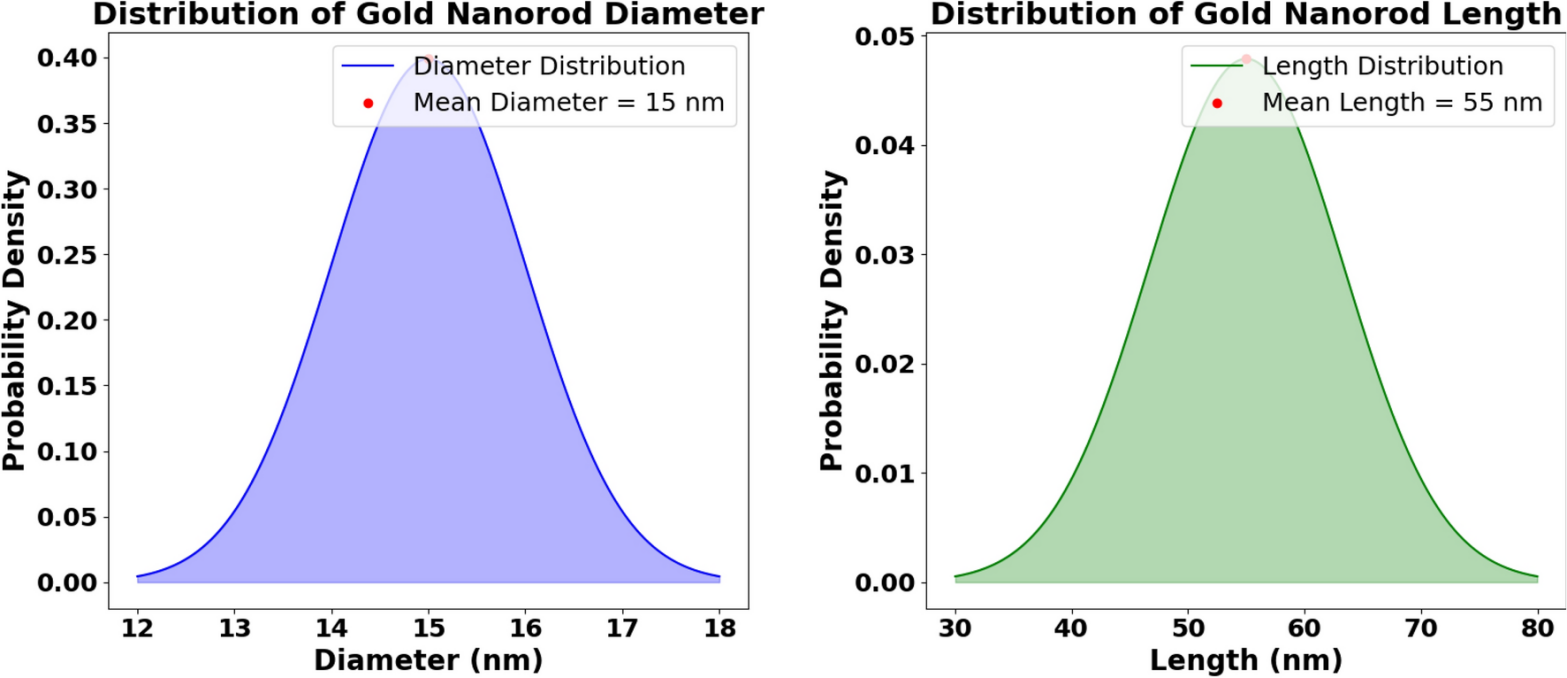
Size distribution for experimental used sample.

### Heat generation source considering size distribution

To account for the variability in the dimensions of AuNRs and overcome the limitations of experimental samples, as highlighted by Qin and coworkers^56^, we define the heat generation source by integrating over their size distribution (see Fig. 2). The mean dimensions of the AuNRs are denoted as $\mu _{d_s}$ for the mean diameter and $\mu _{d_l}$ for the mean length, while $\sigma _{d_s}$ and $\sigma _{d_l}$ represent the standard deviations of the diameter and length distributions, respectively. The variability in dimensions is modeled using normal distributions for both diameter and length.

We denote the heat generation function by $S_e(d_s, d_l)$ and introduce an integrand function, $I(d_s, d_l)$, for the double integration. Using Eq. (25), the integrand function $I(d_s, d_l, \mu _{d_s}, \sigma _{d_s}, \mu _{d_l}, \sigma _{d_l})$ is defined as:

26 $$\begin{aligned} I(d_s, d_l, \mu _{d_s}, \sigma _{d_s}, \mu _{d_l}, \sigma _{d_l}) = S_e(d_s, d_l) \cdot \text {PDF}_{d_s}(d_s) \cdot \text {PDF}_{d_l}(d_l) \end{aligned}$$

Here, $\text {PDF}_{d_s}(d_s)$ and $\text {PDF}_{d_l}(d_l)$ are the probability density functions for $d_s$ (diameter) and $d_l$ (length), modeled as normal distributions with the respective means and standard deviations:

27 $$\begin{aligned} & \text {PDF}_{d_s}(d_s) = \frac{1}{\sigma _{d_s} \sqrt{2\pi }} \exp \left( -\frac{(d_s - \mu _{d_s})^2}{2\sigma _{d_s}^2}\right) \end{aligned}$$

28 $$\begin{aligned} & \text {PDF}_{d_l}(d_l) = \frac{1}{\sigma _{d_l} \sqrt{2\pi }} \exp \left( -\frac{(d_l - \mu _{d_l})^2}{2\sigma _{d_l}^2}\right) \end{aligned}$$

To ensure comprehensive coverage of the size distribution, the integration bounds are set to $\pm 3$ standard deviations from their respective means. This approach captures approximately 97% of the data within the distribution:

29 $$\begin{aligned} d_{s,\text {min}}&= \mu _{d_s} - 3\sigma _{d_s},&d_{s,\text {max}}&= \mu _{d_s} + 3\sigma _{d_s} \end{aligned}$$

30 $$\begin{aligned} d_{l,\text {min}}&= \mu _{d_l} - 3\sigma _{d_l},&d_{l,\text {max}}&= \mu _{d_l} + 3\sigma _{d_l} \end{aligned}$$

The total expected heat generation for AuNRs, $H_{\text {total}}$, is then calculated by integrating the heat generation over the size distribution as follows:

31 $$\begin{aligned} H_{\text {total}}(d_s,d_l) = \int _{d_{l,\text {min}}}^{d_{l,\text {max}}} \int _{d_{s,\text {min}}}^{d_{s,\text {max}}} I(d_s, d_l, \mu _{d_s}, \sigma _{d_s}, \mu _{d_l}, \sigma _{d_l}) \, dd_s \, dd_l \end{aligned}$$

**Figure 3:**
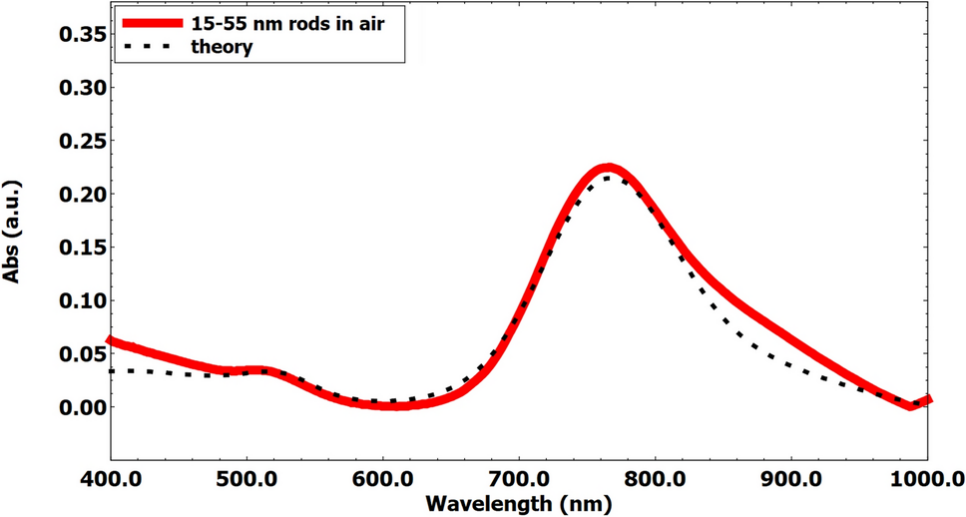
Theoretical^61,62^ absorption spectrum (dashed line) and experimental absorption spectrum (red line) of the AuNR array.

## Conversion model validation with benchmark case

Before performing calculations using the proposed model defined in the previous section, it is appropriate to verify its validity across different incident wavelengths. To this end, Fig. 3 shows the comparison between the absorption of AuNRs over incident wavelengths calculated theoretically and obtained from experimental spectroscopy (Spectrophotometer Thermo Fisher Scientific Evolution 220)^61^. As can be seen in this figure, there is a good agreement for the entire range of incident wavelengths. This validation step ensures that the theoretical model accurately predicts the absorption properties of AuNPs.

To ensure the accuracy of the photothermal conversion model for AuNPs under laser irradiation, a second validation study was conducted. This step was aimed at validating the way the theoretical model was integrated into CFD calculations. The benchmark case involved NPs with specific geometric dimensions, irradiated by a laser at a wavelength of 808 nm, which is commonly used for photothermal applications due to its deep tissue penetration and efficient absorption by AuNRs. The results demonstrated the model’s capability to accurately predict the absorption properties and subsequent heat generation, thereby validating its use for further optimization and analysis.

### Experimental procedure

The experimental procedure involved the use of an 808 nm wavelength laser, selected for its relevance in PTT, with a power of 0.8 W. The AuNRs used had a mean diameter of $\mu _{d_s}$ = 15 nm and a mean length of $\mu _{d_l}$ = 55 nm, with standard deviations of $\sigma _{d_s}$ = ±1 nm for diameter and $\sigma _{d_l}$ = ±8.33 nm for length. The AuNRs were deposited on a glass substrate using the successive-layer assembly technique, enabling them to be immobilized on both sides of the substrate. Next, a borosilicate glass double cell was prepared by strictly following the procedure described in reference^15^. Further details are available in reference^24,83,84^.

**Figure 4:**
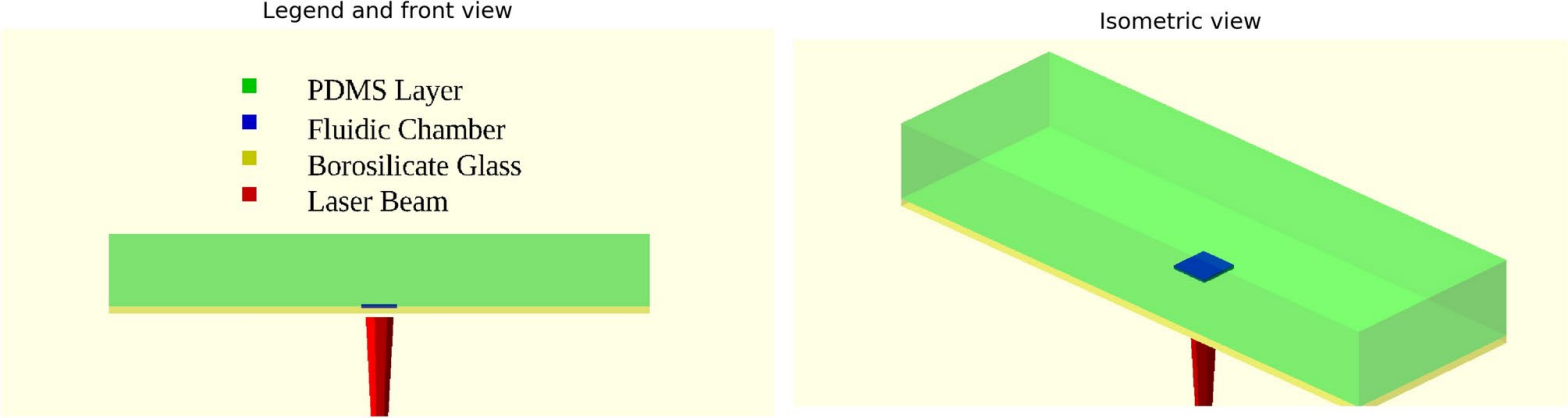
Physical model used for energy conversion model validation.

### Theoretical procedure

**Table 1:** Parameters used in the heat generation model related to experiment.

Parameter	Value	Parameter	Value
Wavelength ($\lambda$)	$808$ nm	Laser power ($P_{n0}$)	0.8 W
Borosilicate refractive index ($n_{\text {Bororef}}$)	1.51062	Occupancy factor ($OF$)	1/3
Air refractive index ($n_{\text {Airref}}$)	1.00028	Beam diameter ($dB$)	0.001 m
Water refractive index ($n_{\text {Waterref}}$)	1.32808	Capping agent thickness ($d_{\text {CT}}$)	$3.8 \times 10^{-9}$ m
Capping agent refractive index ($n_{\text {ctab}}$=$n_{\text {sh}}$)	1.52056	Distance beetween NPs ($\phi$)	$78.8 \times 10^{-9}$ m
Gold refractive index ($n_{\text {Au}}$)	0.155814	Mean length ($\mu _{d_l}$)	55 nm
Gold extinction coefficient ($k_{\text {Au}}$)	4.98973	Standard deviation length ($\sigma _{d_l}$)	8.33 nm
Gold refractive index real ($pr_{\text {Au}}$)	-24.8731	Mean diameter ($\mu _{d_s}$)	15 nm
Gold refractive index imaginary ($pi_{\text {Au}}$)	1.55494	Standard deviation diameter ($\sigma _{d_s}$)	1 nm
Complex refractive index ($ref_{\text {Au}}$)	$pr_{\text {Au}} + i \cdot pi_{\text {Au}}$		

#### CFD calibration and calculations

Using the specified AuNRs dimensions and laser parameters, we calculated the total heat generation rate ($H_{\text {total}}(d_s,d_l)$) using parameters defined in Table 1. And, the size distribution of the AuNRs was presented as distribution functions for both diameter and length (see Fig. 2). After determining the generated heat by the benchmark sample of AuNPs using the proposed conversion model, the generated heat is used as a boundary condition in the performed CFD simulation.

The device used for the validation of the model comes from Sapienza laboratory experiment^83,84^. Indeed, the geometry consists of a thin fluidity chamber with a thickness of 10 $\upmu$m, constructed by attaching a borosilicate glass plate (thickness of 1 mm) isolated by a PolyDiMethylSiloxane (PDMS) plate (thickness of 1 cm). AuNPs, modeled as a thin film with a thickness of $l_{\text {shAu}}$, were embedded at the chamber’s bottom. The device was subjected to a laser beam, as detailed in Figure 4, which provides setup specifics.

#### Boundary conditions

Boundary conditions that should reflect the real-world scenario. In the CFD simulation using ANSYS Fluent software, the interfaces between solid-solid and fluid-solid were automatically set to the heat flux continuity condition. This condition can be described as follows:

32 $$\begin{aligned} & \frac{\partial T_{\text {s1}}}{\partial \textbf{n}} = \frac{k_\text {s2}}{k_\text {s1}} \frac{\partial T_{\text {s2}}}{\partial \textbf{n}}, \quad T_{\text {s1}} = T_{\text {s2}} \end{aligned}$$

33 $$\begin{aligned} & \frac{\partial T_{\text {f}}}{\partial \textbf{n}} = \frac{k_\text {s}}{k_\text {f}} \frac{\partial T_{\text {s}}}{\partial \textbf{n}}, \quad T_{\text {f}} = T_{\text {s}} \end{aligned}$$

34 $$\begin{aligned} & H_{\text {total}}(d_s,d_l) = \frac{\partial T_{\text {f}}}{\partial \textbf{n}} = \frac{k_\text {Au}}{k_\text {f}} \frac{\partial T_{\text {Au}}}{\partial \textbf{n}}, \quad T_{\text {f}} = T_{\text {Au}} \end{aligned}$$

Here, $\textbf{n}$ represents the normal vector, and the following terms are defined:

- $T_{\text {s1}}$: Temperature at the first solid material (borosilicate glass).
- $T_{\text {s2}}$: Temperature at the second solid material (PDMS).
- $T_{\text {s}}$: Temperature at the solid material in contact with the fluid.
- $T_{\text {f}}$: Temperature of the fluid.
- $T_{\text {Au}}$: Temperature of the AuNPs.
- $k_\text {s1}$: Thermal conductivity of the first solid material (borosilicate glass).
- $k_\text {s2}$: Thermal conductivity of the second solid material (PDMS).
- $k_\text {s}$: Thermal conductivity of the solid material in contact with the fluid.
- $k_\text {f}$: Thermal conductivity of the fluid.
- $k_\text {Au}$: Thermal conductivity of AuNPs.

Due to the presence of the capping agent material that covers the AuNPs, the Marangoni stress condition at the contact surface between the gold AuNPs slab and the fluid was applied. The Marangoni stress can be expressed by the following equation:

35 $$\begin{aligned} \sigma _{\text {Marangoni}} = -\gamma ' \nabla T \end{aligned}$$

Where $\sigma _{\text {Marangoni}}$ represents the Marangoni stress, $\gamma '$ means the gradient of surface tension with respect to temperature, and $\nabla T$ represent the temperature gradient along the interface.

The hydrodynamic condition at the other fluid/solid interfaces is maintained at a no-slip condition. In addition, the bottom surface of the glass was subjected to mixed convection and radiation boundary conditions. This combination ensures a realistic simulation environment by accounting for both heat transfer mechanisms. The heat flux is expressed as:

36 $$\begin{aligned} \dot{Q} = h_\text {ext} \cdot (T_{\infty } - T_{w}) + \epsilon \cdot \sigma _\text {SB}\ \cdot (T_{\text {ext}}^{4} - T_{w}^{4}) \end{aligned}$$

where:

- $\sigma _\text {SB}$: Stefan–Boltzmann constant ($5.67 \times 10^{-8} \, \text {W} \cdot \text {m}^{-2} \cdot \text {K}^{-4}$),
- $\epsilon$: Emissivity (dimensionless, typically between 0 and 1),
- $h_\text {ext}$: Heat transfer coefficient ($\text {W} \cdot \text {m}^{-2} \cdot \text {K}^{-1}$),
- $$T_{\infty }$$: Ambient temperature (K),
- $T_{w}$: Surface temperature of the wall (K),
- $T_{\text {ext}}$: External environment temperature (K).

**Figure 5:**
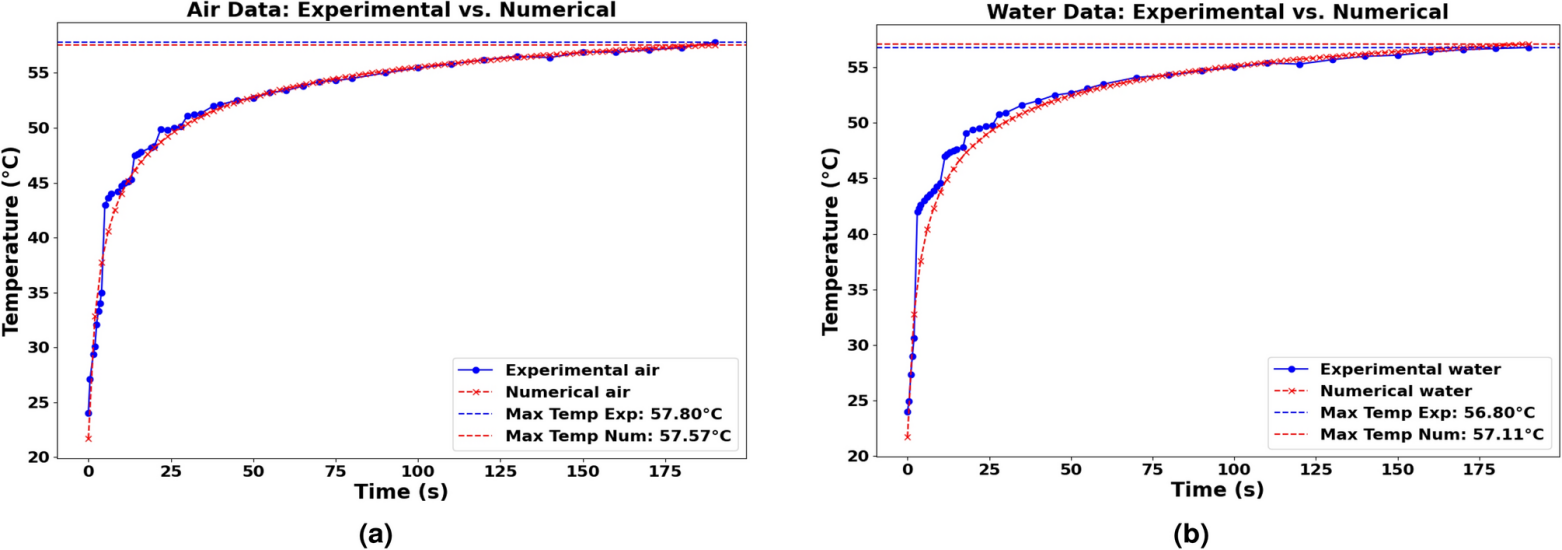
Energy conversion model validation against experimental results for air (**a**) and water (**b**) case data comparison.

#### CFD setup and simulation

The problem considered here involves conjugate heat transfer, where heat is transported simultaneously in both solid and fluid mediums. ANSYS Fluent commercial software employs the finite volume method to model these heat transfer processes and to solve the Navier-Stokes equations for fluid flow alongside the energy equation for both solid and fluid domains. To manage the setup of the software and accurately set the boundary conditions at the gold slab surface, a User-Defined Function (UDF) was developed in C program. This UDF, based on the DEFINE_PROFILE macro, performs a systematic scan of the slab gold surface and selects a $3\times 3$ mm$^{2}$ area that corresponds to the beam size of the laser used in the experiment. This targeted approach ensures that heat generation is accurately modeled in the region most affected by the laser.

The computational model used in Ansys Fluent software utilizes the SIMPLE algorithm, which can handles the pressure-velocity coupling in the flow filed. The second-order upwind scheme was used for spatial discretization of the convective terms in the momentum and energy equations. A non-iterative first-order scheme with a fixed time step of (0.0002 s) was used to descretize the transient terms up to *t* = 190 s. The structured rectangular mesh grid was generated with 15 million element, which was found good to have relatively quick convergence time and good fit of the result with experimental data. The simulations were conducted using ANSYS Fluent (v23 R1, ANSYS Inc.)^85^, and the calculation are run in the TrytonPlus supercomputer, which has 72 cores.

####  Comparison and validation

The microchamber was filled sequentially with water and air in separate simulations to assess the impact of the surrounding medium on heat transfer and fluid flow. These simulations aimed to compare the temperature-time dependence during irradiation and the temperature fields against experimental data. The validation process involved comparing numerical simulation results with experimental observations. This comparison focused on the temporal temperature profiles (see Fig. 5a, b) within the microchamber under irradiation. As it was shown in validation figures, namely Figs. 3, 5a, b, a good agreement between the numerical and experimental data would validate the accuracy of our photothermal conversion model and the effectiveness of AuNRs as a heat source in microfluidic devices.

**Figure 6:**
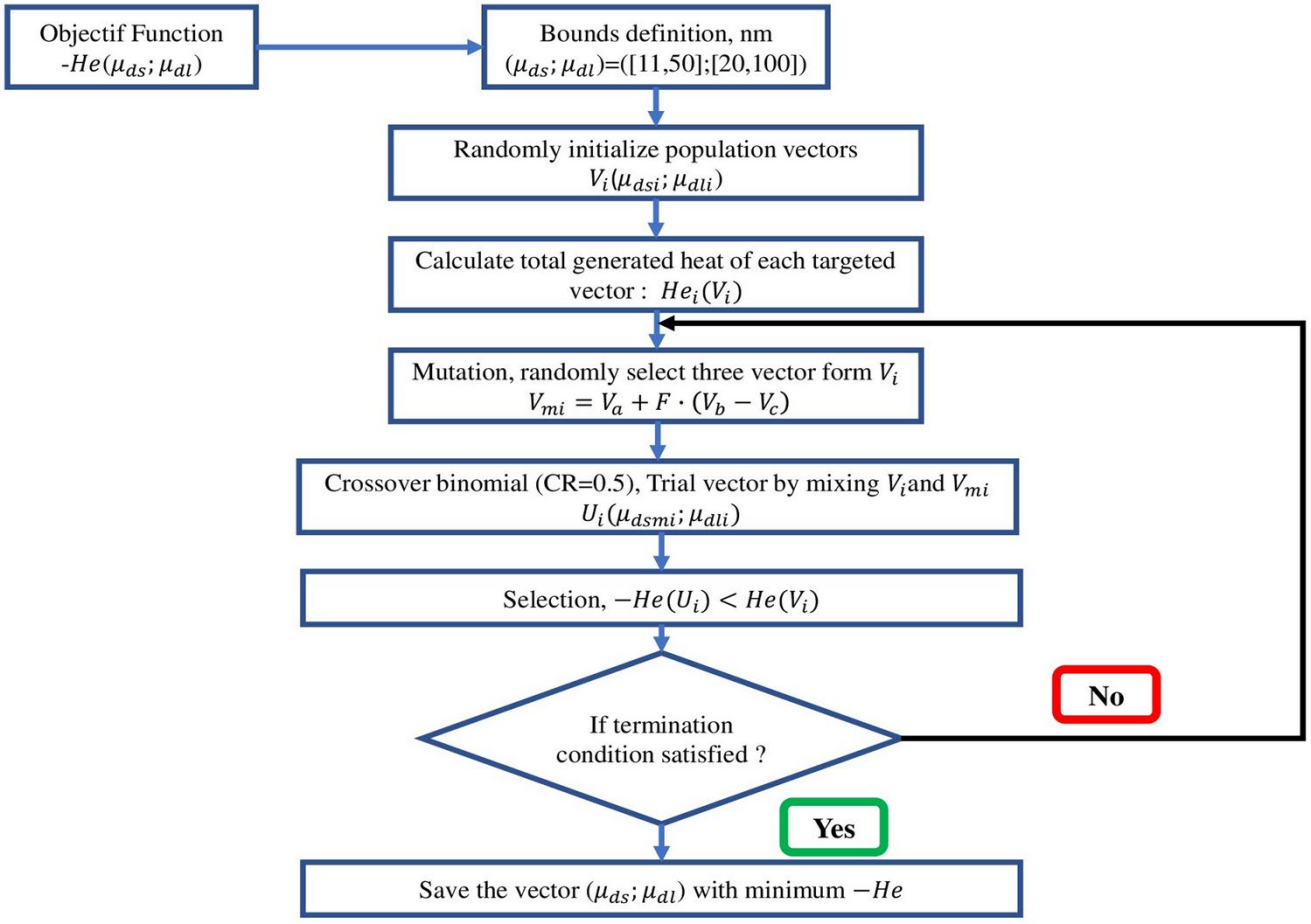
Flowchart of DE algorithm for the optimization of the size and shape of AuNRs for heat generation.

## Optimization via differential evolution algorithm

In this work, the DE algorithm, introduced by Storn and Price in 1997^86^, was used for the optimization process. DE is recognized as a robust and versatile optimization method, particularly effective for high-dimensional and nonlinear problems^87^. It is well-suited for optimizing complex objective functions, such as the heat generation function $H_{\text {total}}(d_s, d_l)$, derived from the light absorption properties of AuNRs. Unlike gradient-based methods, DE does not require derivative information, making it ideal for optimizing non-differentiable or noisy functions^88^.

The DE algorithm iteratively improves a population of candidate solutions based on a given objective function. In this study, DE was utilized to maximize the total heat generation $H_{\text {total}}(d_s, d_l)$, effectively searching the solution space and avoiding local optima through its unique mutation and crossover strategies, which balance exploration and exploitation.

Compared to other optimization techniques, such as genetic algorithms (GA) or particle swarm optimization (PSO), DE offers several advantages^87,89^:

- Simplicity: DE uses fewer parameters and is easier to implement than GA or PSO, reducing computational overhead^90^.
- Global optimization: DE is effective at avoiding local optima, ensuring convergence toward a global solution. Hence, it should be emphasis that the deterministic algorithms (e.g. Implicit Filtering; Nelder-Mead and Pattern Search) are characterised with possibility of falling into the area of local minimum^91–94^. However, the integrative hybrid Nelder-Mead and particle swarm optimization could be attributed to its versatility of usage and possibility to solve the complicated task more comprehensively. This method assures the enhance of efficiency in relation to the original case obtained in the whole range of parameters^94,95^.
- No derivative dependency: DE works well with objective functions that are non-differentiable, multi-modal, or noisy^96^.
- Efficiency: The adaptive nature of DE’s mutation factor allows it to converge faster than GA for problems with continuous variables.

These attributes make DE a suitable choice for optimizing the photothermal conversion efficiency of AuNPs. The objective of the optimization process is the find the combination of $(d_{s}, d_{l})$ that maximize the $H_{\text {total}}(d_s, d_l)$, hence the objective function can be expressed as:

37 $$\begin{aligned} \text {Minimize} \quad [-H_{\text {total}}(d_s, d_l)] \end{aligned}$$

Here, the minus sign is used to maximize heat generation.

The DE algorithm, renowned for its efficacy in handling complex optimization problems, progresses through the following stages (see Fig. 6):

- Initialization: A population of candidate solutions, $\textbf{P}$, is generated, where each vector $\textbf{x}_i = (d_{s,i}, d_{l,i})$ represents a set of NRs dimensions.
- Mutation: For each target vector $\textbf{x}_i$, a mutant vector $\textbf{v}_i$ is constructed as:
  38 $$\begin{aligned} \textbf{v}_i = \textbf{x}_{r1} + F \cdot (\textbf{x}_{r2} - \textbf{x}_{r3}), \end{aligned}$$
  where $r1$, $r2$, and $r3$ are distinct indices selected randomly, and $F$ is the differential weight. The mutation factor $F$ can also be adaptive:
  39 $$\begin{aligned} F^{(g)} = F_0 \cdot \left( 1 - \frac{g}{G_{\text {max}}}\right) , \end{aligned}$$
  where $F_0$ is the initial mutation factor, $g$ is the current generation, and $G_{\text {max}}$ is the maximum number of generations.
- Crossover: A trial vector $\textbf{u}_i$ is generated by combining elements of $\textbf{x}_i$ and $\textbf{v}_i$, typically through a binomial crossover:
  40 $$\begin{aligned} u_{ij} = {\left\{ \begin{array}{ll} v_{ij} & \text {if } \text {rand}(j) \le CR \text { or } j = \text {rand}(n) \\ x_{ij} & \text {otherwise} \end{array}\right. }, \end{aligned}$$
  where $CR$ is the crossover probability, and $\text {rand}(n)$ ensures at least one component from $\textbf{v}_i$ is included.
- Selection: The trial vector $\textbf{u}_i$ is evaluated against $\textbf{x}_i$, with the one yielding a higher $H_{\text {total}}$ value retained for the next generation:
  41 $$\begin{aligned} \textbf{x}_i^{(g+1)} = {\left\{ \begin{array}{ll} \textbf{u}_i & \text {if } H_{\text {total}}(\textbf{u}_i) > H_{\text {total}}(\textbf{x}_i) \\ \textbf{x}_i & \text {otherwise} \end{array}\right. }. \end{aligned}$$
- Convergence: The algorithm continues iterating until the change in the objective function satisfies:
  42 $$\begin{aligned} \left| H_{\text {total}}^{(g+1)} - H_{\text {total}}^{(g)} \right| < \epsilon , \end{aligned}$$
  where $\epsilon$ is a small positive threshold.
- Population diversity: The diversity of the population is measured by:
  43 $$\begin{aligned} D^{(g)} = \frac{1}{N} \sum _{i=1}^{N} \Vert \textbf{x}_i^{(g)} - \bar{\textbf{x}}^{(g)}\Vert , \end{aligned}$$
  where $D^{(g)}$ is the diversity at iteration $g$, $N$ is the population size, and $\bar{\textbf{x}}^{(g)}$ is the mean vector of the population.

**Figure 7:**
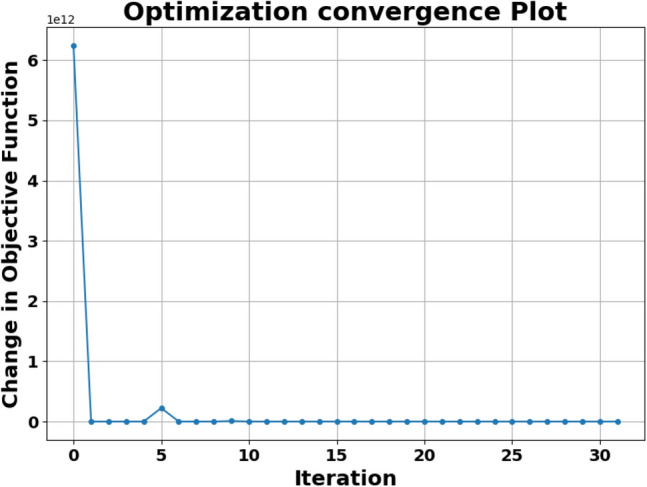
Optimization convergence plot for $\lambda = 808$ nm.

**Figure 8:**
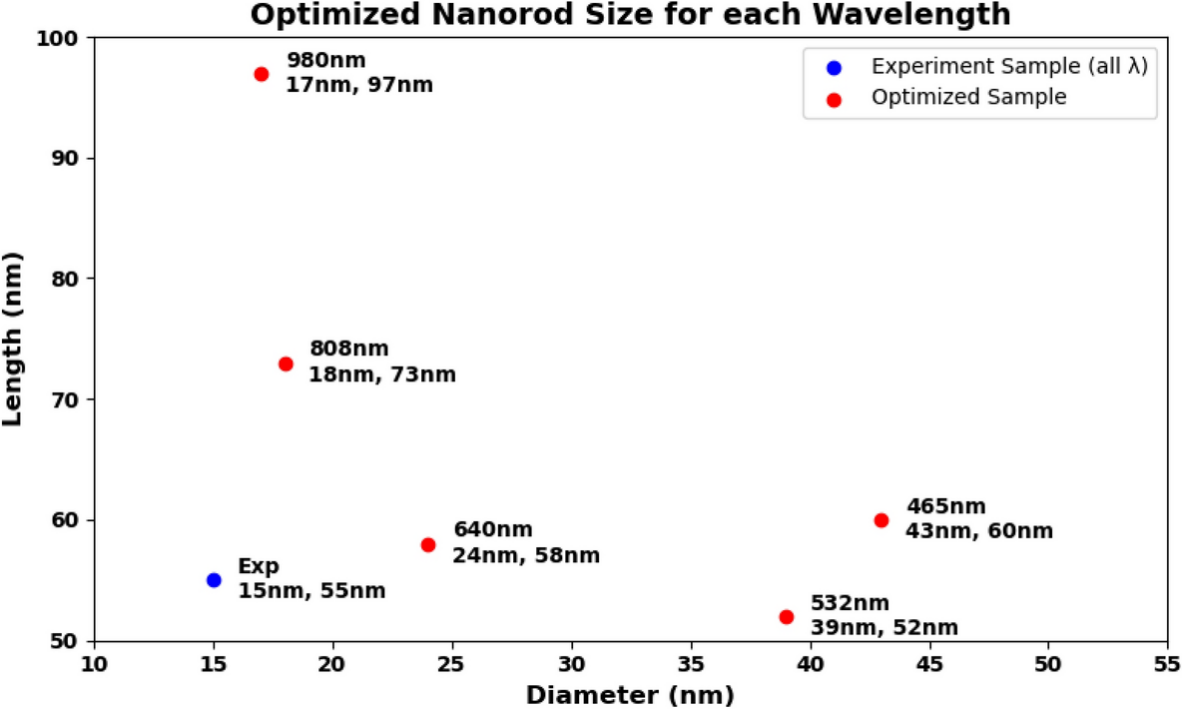
Optimized AuNRs illustrated by length and diameter results for each wavelength.

The convergence of the optimization algorithm during iteration is given in Fig. 7 for wavelength $\lambda = 808$ nm. This figure illustrates the change in the objective function during successive iterations of the DE algorithm. Initially, the graph shows a significant change, indicating significant improvements in the value of the objective function as the algorithm explores the solution space. This evolution is followed by a rapid decrease in the variation of the objective function, indicating that the algorithm is rapidly approaching an optimal solution. After the first few iterations, the variation stabilizes near zero, indicating convergence and that subsequent iterations bring only minimal improvements.

## Optimization results

In this section, the results connected to the optimization of the size and shape of AuNRs to maximize heat generation when irradiated by different wavelengths have been presented. The selected wavelengths for optimization were 465 nm, 532 nm, 640 nm, 808 nm, and 980 nm. Hence, these wavelengths cover a broad spectrum, including visible to NIR regions, which are relevant for various photothermal applications.

**Figure 9:**
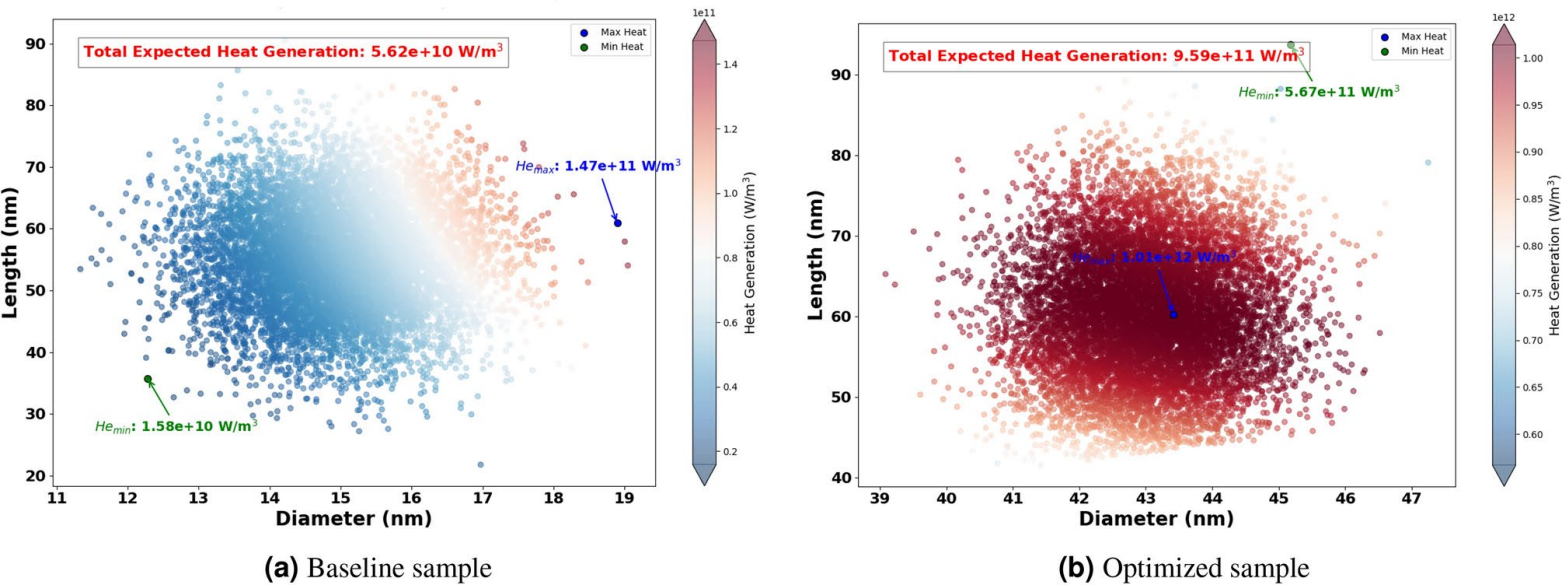
Expected heat generation with baseline (**a**) vs optimized (**b**) sample irradiated by $\lambda = 465$ nm.

**Figure 10:**
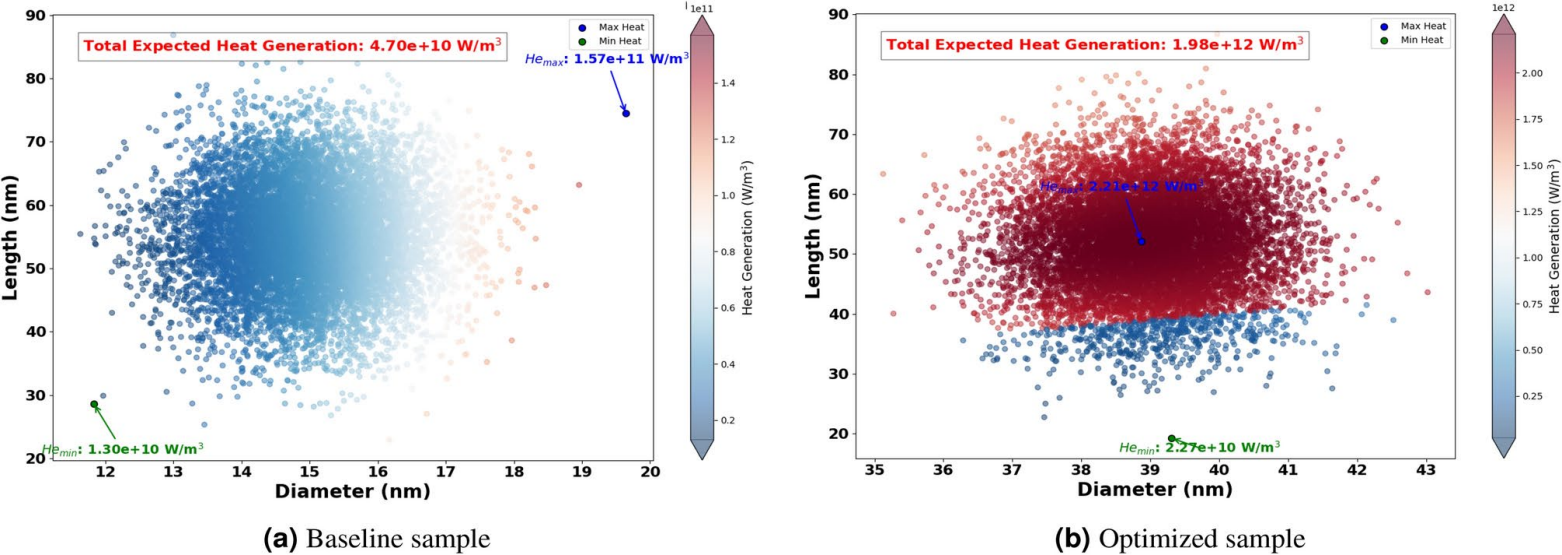
Expected heat generation with baseline (**a**) vs optimized (**b**) sample irradiated by $\lambda = 532$ nm.

The Fig. 8 shows the optimized dimensions of AuNRs using different wavelengths, showcasing both diameter and length. Where, the red dots represent the optimized AuNPs dimensions for specific wavelengths (465 nm, 532 nm, 640 nm, 808 nm, and 980 nm), while the blue dot indicates the dimensions of the experimental sample. From this figure, it can be seen clearly that the wavelength has a significant impact on the optimized dimensions of the AuNPs. For example, at 465 nm and 532 nm, the AuNPs have a near spherical shape with dimensions of (43 nm, 60 nm) and (39 nm, 52 nm) successively. This suggest that shorter light wavelength resonate more effectively with more symmetrical AuNPs. Indeed, this observation aligns with the findings by Link and El-Sayed^97^, who noted strong plasmonic resonance in smaller, symmetrical AuNPs due to their efficient light absorption properties. In another hand, for longer wavelengths such as 808 nm and 980 nm, elongated shapes such as NRs have found optimal. This is because longer light wavelengths interact better with particles that have higher inverse AR, such as NRs , which can support plasmonic resonances along their longer axis, as highlighted in studies by^98,99^.

**Figure 11:**
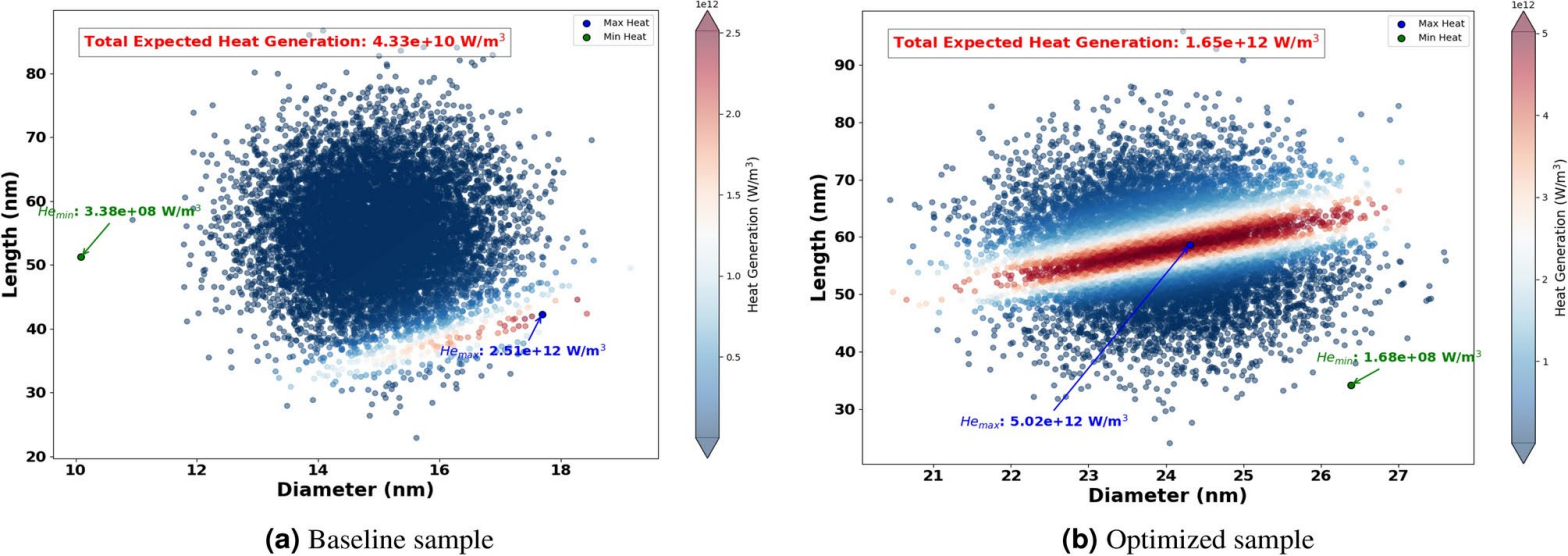
Expected heat generation with baseline (**a**) vs optimized (**b**) sample irradiated by $\lambda = 640$ nm.

**Figure 12:**
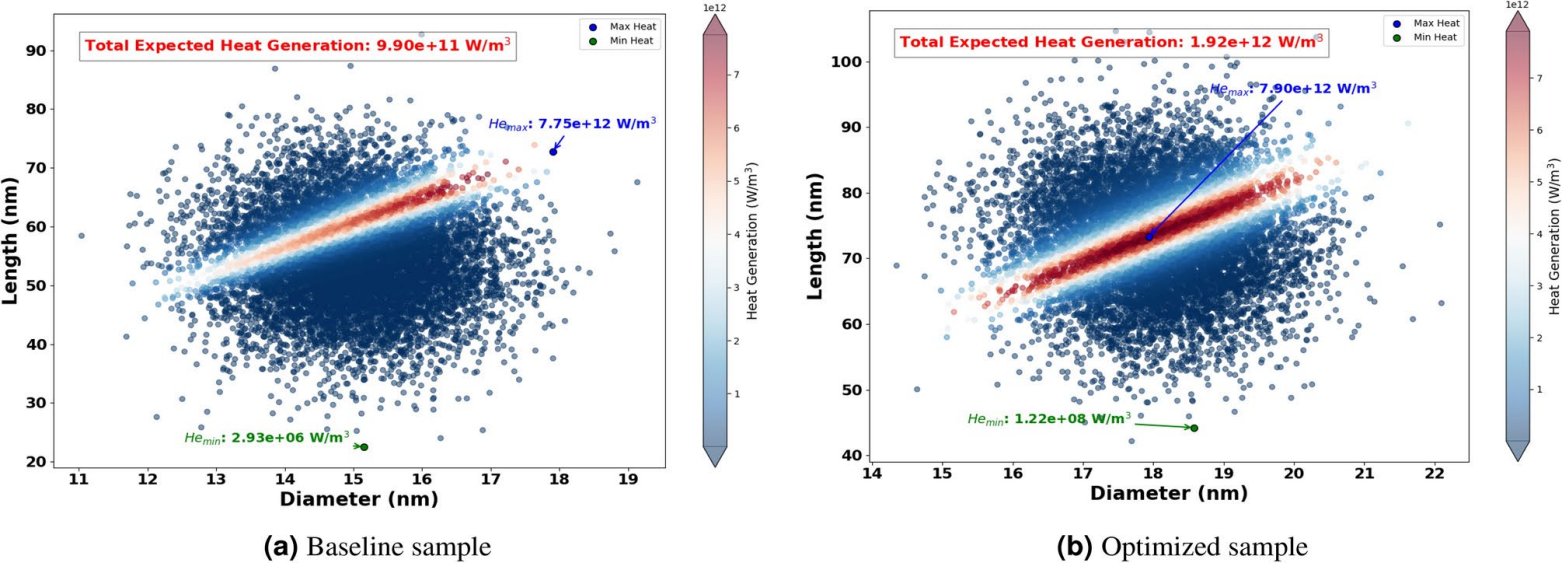
Expected heat generation with baseline (**a**) vs optimized (**b**) sample irradiated by $\lambda = 808$ nm.

**Figure 13:**
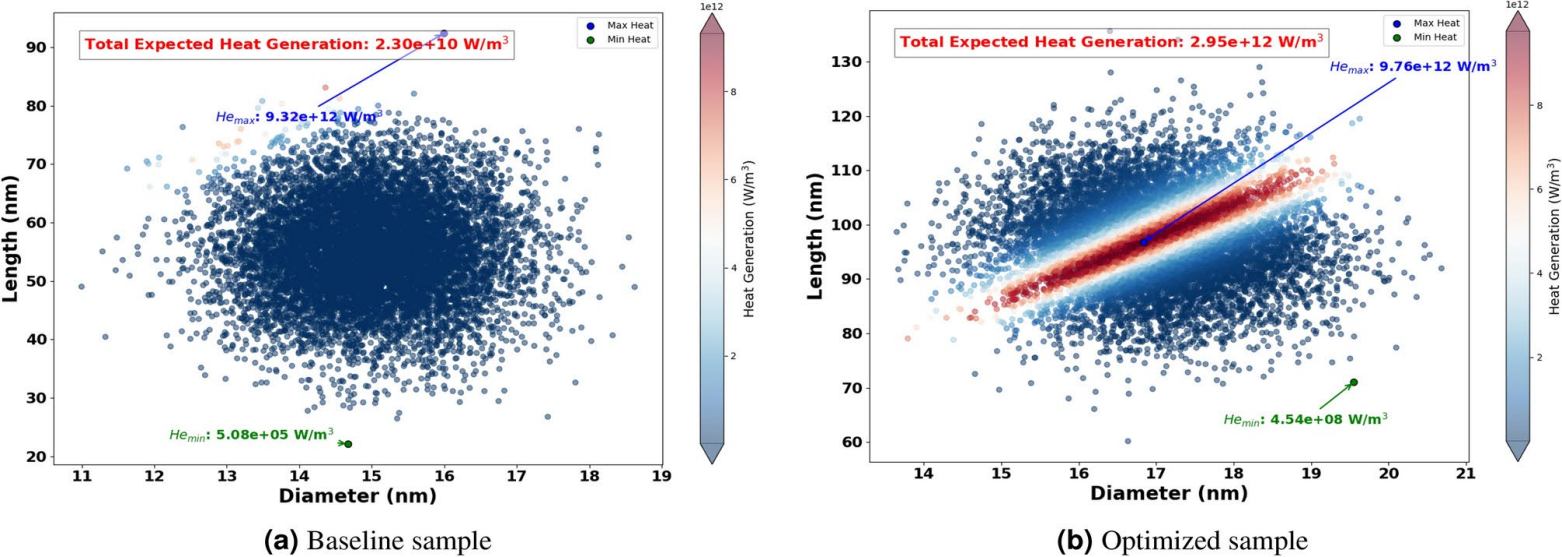
Expected heat generation with baseline (**a**) vs optimized (**b**) sample irradiated by $\lambda = 908$ nm.

The optimization process identified the optimum size and shape of AuNRs for varied wavelengths (see Fig. 8 ). Using these optimized parameters, the expected heat generation results for the baseline and optimized samples at different wavelengths (465 nm, 532 nm, 640 nm, 808 nm and 980 nm) were presented. As shown in the results from Figs. 9, 10, 11, 12 and 13, it can be clearly demonstrate that the optimization process significantly enhances the heat generation capabilities of AuNPs across different wavelengths.

Relatively to the short wavelengths (465 nm and 532 nm), in Figs. 9 and 10, a comparison of the expected heat generation and the heat generation of individual NRs using experimental and optimized samples has been presented. In both cases, the baseline samples show wide and dispersed heat generation values, indicating inefficient AuNRs dimensions. In the baseline samples, the heat generation values are very scattered, which indicates a wide range of AuNRs dimensions that are not well matched to maximize heat generation at their respective wavelengths. Indeed, the baseline heat generation for 465 nm ranges from $1.58 \times 10^{10} \text { W/m}^3$ to $1.47 \times 10^{11} \text { W/m}^3$, while for 532 nm it ranges from approximately $1.30 \times 10^{10} \text { W/m}^3$ to $1.57 \times 10^{11} \text { W/m}^3$. This wide dispersion indicates inefficient heat generation due to sub-optimal AuNRs dimensions.

**Figure 14:**
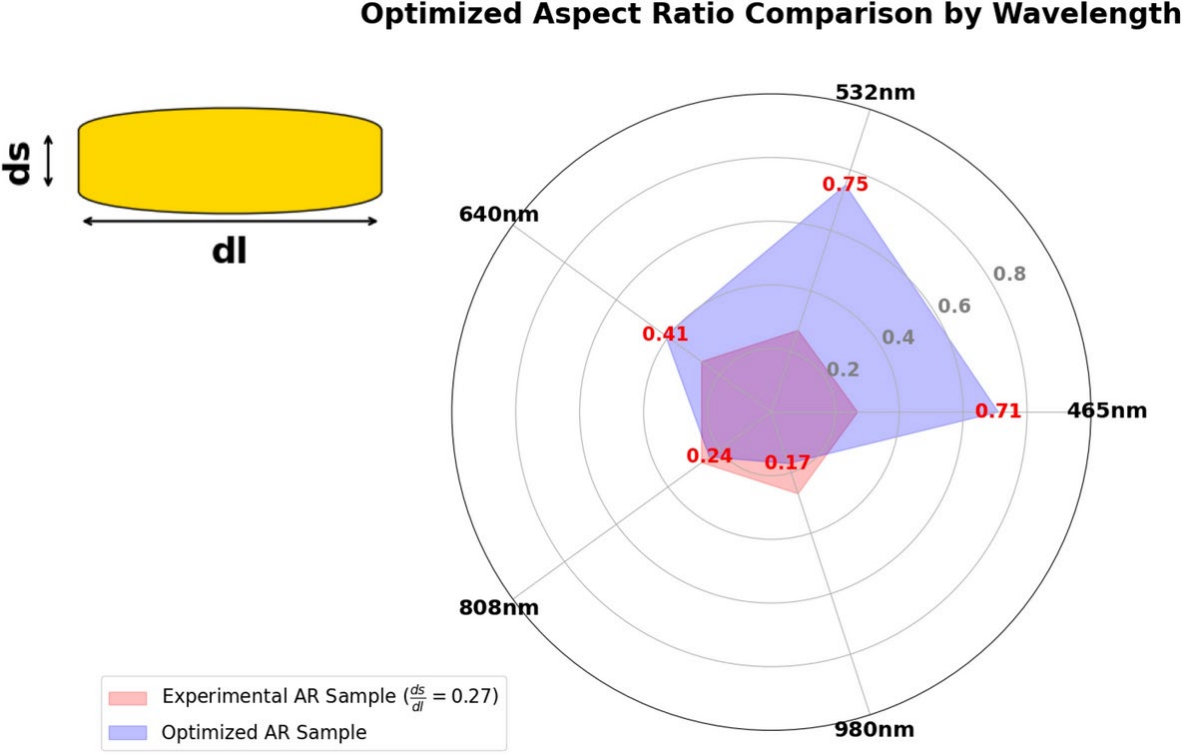
Radar presentation of the optimized AR for different wavelength: comparison with the experimental AR.

**Figure 15:**
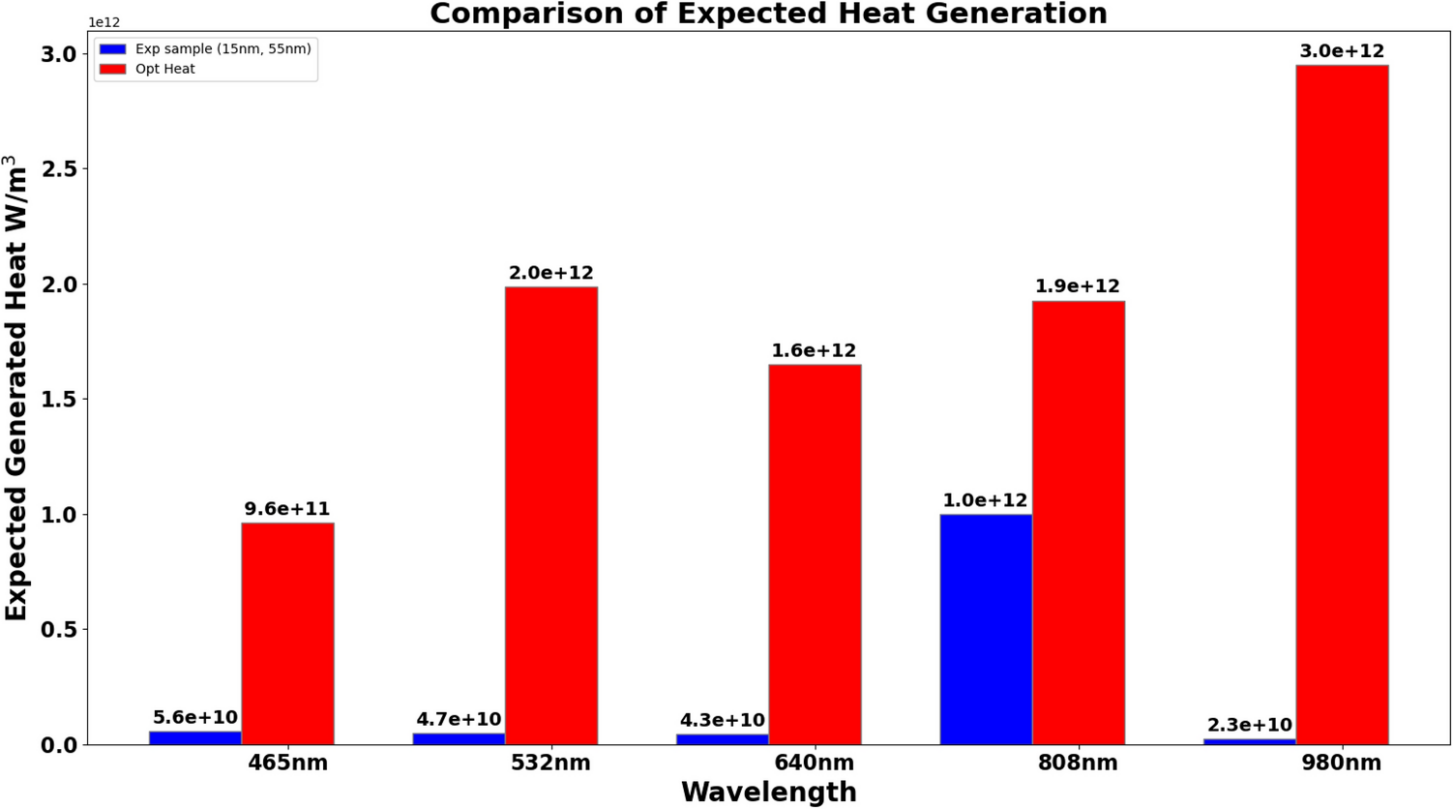
Expected heat generation using experimental sample comparison with optimized sample of AuNRs.

To summarize the optimization results, we present the radar chart that compares the AR of the experimental and optimized samples of AuNRs across different wavelengths (see Fig. 14). As shown in this figure, the AR was increased to 0.71 from the experimental value of 0.27, indicating a preference for more near-spherical particles when using shorter wavelengths. In the NIR, the AR was found to decrease from 0.27 to 0.24 and 0.17, indicating that more elongated NRs were more effective at these longer wavelengths. As a result, the bar chart in Fig. 15 illustrates the expected heat generation for both experimental and optimized AuNRs. Significant improvements can be drawn from these bars when using optimized AuNRs derived from our results. Specifically, at 465 nm, the expected heat generation increased from $5.6 \times 10^{10} \, \text {W/m}^3$ in the experimental sample to $9.6 \times 10^{11} \, \text {W/m}^3$ in the optimized sample. At 532 nm, the heat generation increased from $4.7 \times 10^{10} \, \text {W/m}^3$ to $2.0 \times 10^{12} \, \text {W/m}^3$. For the 640 nm wavelength, the heat generation rose from $4.3 \times 10^{10} \, \text {W/m}^3$ to $1.6 \times 10^{12} \, \text {W/m}^3$. In addition, at 808 nm, it increased from $1.0 \times 10^{12} \, \text {W/m}^3$ in the experimental sample to $1.9 \times 10^{12} \, \text {W/m}^3$ in the optimized sample. Finally, at 980 nm, the expected heat generation significantly increased from $2.3 \times 10^{10} \, \text {W/m}^3$ to $3.0 \times 10^{12} \, \text {W/m}^3$. This demonstrates the effectiveness of the optimization process in enhancing the heat generation capabilities of AuNRs across different wavelengths.

## Case study

**Figure 16:**
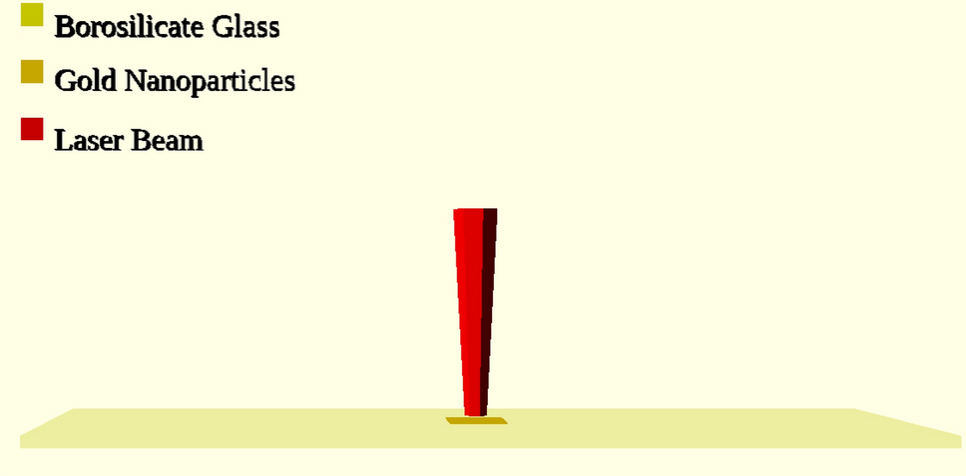
Borosilicate glass plate irradiated by laser with an overview diagram used in the case study.

**Figure 17:**
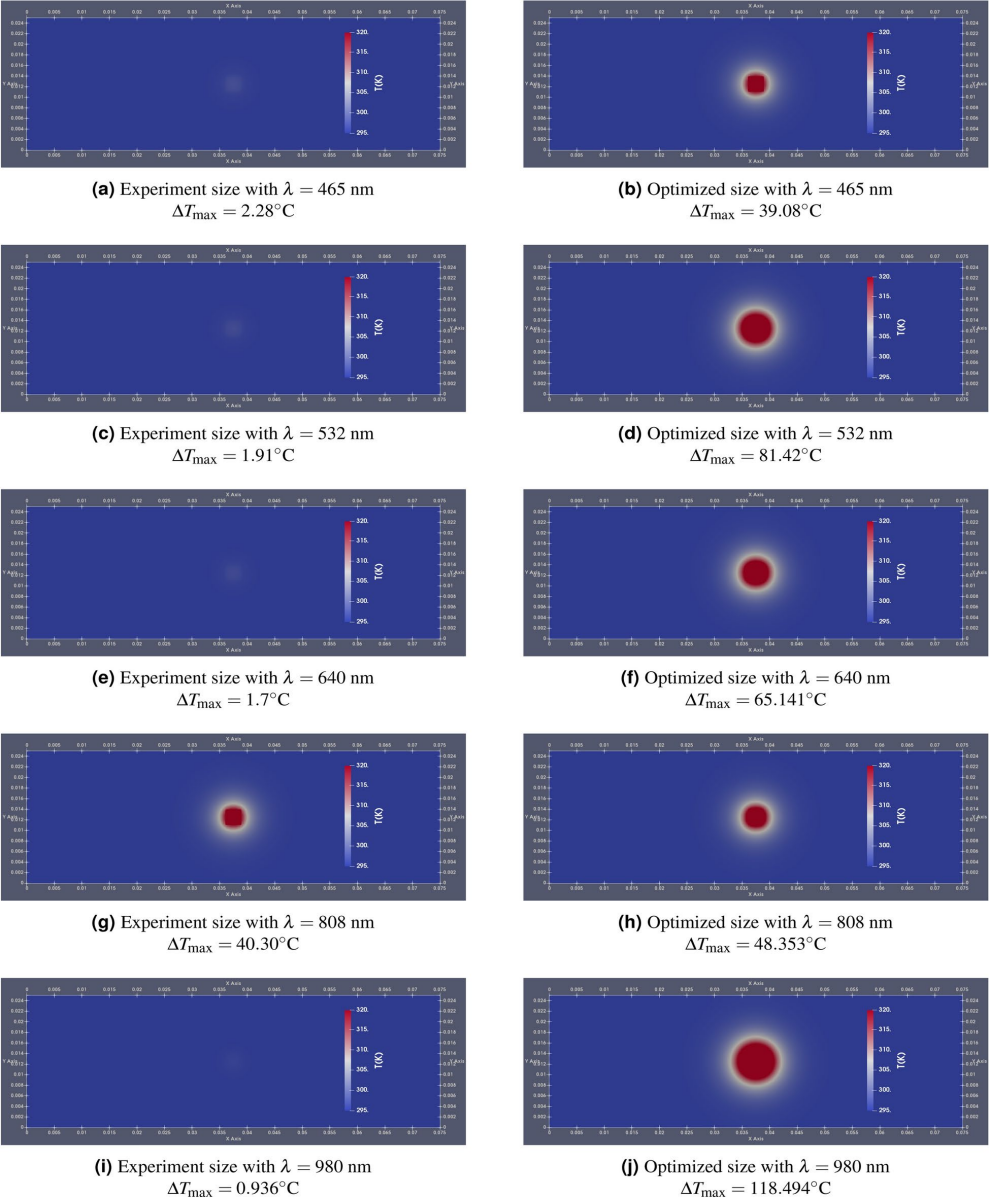
CFD calculations using the experimental AuNRs sizes (15 nm diameter, 55 nm length) for different laser wavelengths (left: (**a**), (**c**), (**e**), (**g**), (**i**)) vs CFD calculations using the optimized AuNRs sizes determined for each specific wavelength (right: (**b**), (**d**), (**f**), (**h**), (**j**)).

After validating the theoretical model and the optimization procedure for determining the optimal size and shape of AuNRs at each wavelength, a practical case study reflecting real-world conditions was explored in this section. The case study involves a borosilicate glass plate with dimensions of 25 mm by 75 mm by 1 mm, where a layer of AuNRs is deposited at the center and irradiated by a laser (see Fig. 16). This scenario is modeled using the transient conduction heat transfer equation (Eq. 44) with mixed convection and radiation boundary conditions (Eq. 36). The heat source is treated as a boundary condition at the center of the borosilicate glass plate, utilizing the pre-calculated heat generation values obtained in the previous sections (see Fig. 15). The governing equation for this case can be defined as follows:

44 $$\begin{aligned} \rho c_p \frac{\partial T}{\partial t} = \nabla \cdot (k \nabla T) \end{aligned}$$

where $\rho$ represents the density, $c_p$ defines the specific heat capacity, $k$ is the thermal conductivity, $T$ means the temperature, and $t$ is time. Numerical simulations for this case study were carried out up to $t = 120$ s, using all the wavelengths applied in the optimization section. Therefore, we simulated the case using both samples of AuNRs: experimental and optimized. The aim is to see how temperature evolution can be improved using the new optimized size of AuNRs when deposited on a borosilicate glass surface. In Fig. 17, the comparative evaluation between the effects of the two samples on the temperature field at the glass surface is presented. Here, the scale has been chosen so that the temperature difference is $\Delta T \ge 25^\circ \text {C}$ after 120 s. Here, the experimental AuNRs sizes (15 nm diameter, 55 nm length) are fixed to represent the sizes available in the laboratory, while the optimized AuNRs sizes vary with wavelength as determined by the optimization process. This approach highlights the potential improvements in heat generation achievable through tailored AuNR designs for specific wavelengths. As can be seen in this figure, the temperature difference after 120 s shows a different rate of increase depending on the laser wavelength. This states that the effectiveness of AuNRs in absorbing and converting light to heat depends on external conditions such as the wavelength of the light, a phenomenon well documented in scientific literature^64^. Indeed, for example, in the case of $\lambda = 465 \,\text {nm}$, $\Delta T_{\text {max}}$ increases from 2.28$^\circ \text {C}$ using the experimental sample ($\mu _{d_s} = 15 \,\text {nm}, \mu _{d_l} = 55 \,\text {nm}$) to 39.08$^\circ \text {C}$ using the optimized sample ($\mu _{d_s} = 43 \,\text {nm}, \mu _{d_l} = 60 \,\text {nm}$), and in the case of $\lambda = 532 \,\text {nm}$ from 1.91 to 81.42$^\circ \text {C}$. This significant increase can be attributed to LPSR, which maximizes absorption at particular NRs sizes and shapes. It’s also clear that for the case of $\lambda = 808 \,\text {nm}$, the rate of increase in $\Delta T_{\text {max}}$ records the lowest value of all the calculations. This is due to the fact that the sample used in the experiment was initially designed for this wavelength based on previous work in the field, confirming the fidelity of our calculations and validating the initial choice. Furthermore, as can be seen in Figs. 17i and 17j, the optimization process significantly enhances the heat generation capabilities of the AuNRs plate for the NIR laser ($\lambda = 980 \,\text {nm}$). The increased heat generation capacity at this wavelength can be explained by the increased absorption efficiency of the optimized AuNRs, which is well documented in photothermal studies^100^. The temperature difference increases from 0.94$^\circ \text {C}$ to 118.494$^\circ \text {C}$. As a result, the temperature field shows different patterns in all cases. In these maps, the red color represents regions where $\Delta T \ge 25^\circ \text {C}$. Indeed, in the cases where the experimental sample is used, this threshold is only reached for the wavelength ($\lambda = 808 \,\text {nm}$), which is indicated by the absence of red-colored regions on the maps relating to these cases. However, the use of the new optimum size of AuNRs greatly affects the heat distribution on the glass surface and contributes to the enlargement of the surface region to $\Delta T \ge 25^\circ \text {C}$. The associated surface region follows the same order as the value of $\Delta T_{\text {max}}$, i.e.: case with $\lambda = 465 \,\text {nm}$ (see Fig. 17b); case with $\lambda = 808 \,\text {nm}$ (see Fig. 17h); case with $\lambda = 640 \,\text {nm}$ (see Fig. 17f); case with $\lambda = 532 \,\text {nm}$ (see Fig. 17d); case with $\lambda = 980 \,\text {nm}$ (see Fig. 17j).

**Figure 18:**
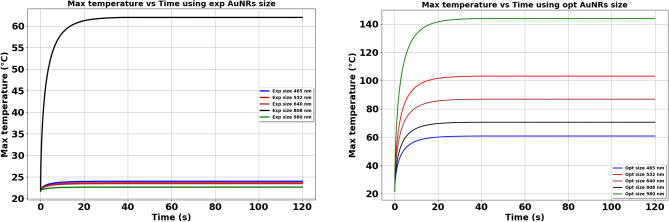
Comparison of the maximum temperature evolution during the time at different laser wavelength, using experimental AuNPs size and optimized AuNPs size.

Figure 18 compares the maximum temperature evolution during the irradiation time in the case of different wavelengths, using both experimental and optimized AuNRs. In the graph on the left, the maximum temperature barely exceeds 25 $^\circ \text {C}$ for all lasers except ($\lambda = 808$ nm), which reaches a maximum of around 60 $^\circ \text {C}$. This confirms the results presented in the temperature field and confirms that the older sizes of AuNRs are not efficient at generating significant heat. In contrast, the graph on the right shows a remarkable increase in $T_{\text {max}}$ using the optimized sizes, regardless of the laser wavelength considered. Here, the maximum temperature reaches around 60 $^\circ \text {C}$ for 465 nm, 102 $^\circ \text {C}$ for 532 nm, 86 $^\circ \text {C}$ for 640 nm, and a remarkable 145 $^\circ \text {C}$ for 980 nm. The significant difference between the two sets of graphs highlights the importance of optimizing AuNRs size for efficient heat generation. Optimized sizes take full advantage of the LSPR effect, enhancing light absorption and conversion to heat. This effect is particularly pronounced at specific wavelengths where resonance conditions are reached, leading to a higher rate of temperature rise.

## Conclusions

In this work, an optimization procedure to determine the optimum diameter and length of AuNRs for maximum heat generation was conducted using a probabilistic DE algorithm. The calculations were based on a theoretical model for light-to-heat conversion, validated against experimental data. A wide range of wavelengths was explored, from $\lambda =465$ nm to $\lambda =980$ nm. The validation procedure involved comparing temperature evolution results collected from CFD simulations using Ansys Fluent software with results collected by a thermal camera during the experimental procedure. The CFD geometry used in this work mirrored the experimental setup, where a fluidic chamber was constructed using borosilicate glass and covered with PDMS.

The first finding was good agreement between the experimental data and the data collected from CFD simulations for both validation tests: the fluidic chamber filled with water and the fluidic chamber filled with air. The optimization results affirmed that the optimal dimensions ($\mu _{d_s}$, $\mu _{d_l}$) strongly depend on the wavelength of the laser used. Indeed, for shorter wavelengths, near-spherical NRs are more favorable for higher heat generation. In contrast, when using longer wavelengths, elongated NRs absorb more light, allowing for greater heat generation. In addition, it is evident that the distance between NRs, which reflects the concentration parameter, influences heat generation; as the distance decreases, the generated heat increases.

After determining the optimum sizes at each wavelength, a case study was conducted that reflects a real-world scenario. This involved the deposition of AuNRs on borosilicate glass, which was then irradiated by lasers of different wavelengths. This case study was conducted by solving the transient conduction heat equation in 3D.

It was found that the temperature difference after 120 s increased at varying rates depending on the laser wavelength. For instance, at $\lambda = 465$ nm, the maximum temperature $T_{\text {max}}$ increased from 2.28 $^\circ \text {C}$ with the experimental sample to 39.08 $^\circ \text {C}$ with the optimized sample. Similarly, at $\lambda = 532$ nm, the temperature increased from 1.91 $^\circ \text {C}$ to 81.42 $^\circ \text {C}$. In contrast, at $\lambda = 808$ nm, the rate of increase in $T_{\text {max}}$ was the lowest due to the initial design of the experimental sample for this wavelength. The optimization process significantly enhanced the heat generation capabilities of the AuNRs plate for the NIR laser ($\lambda = 980$ nm), with the temperature difference increasing from 0.94 to 118.494 $^\circ \text {C}$.

Future efforts will be devoted to experimentally fabricating optimized AuNRs tailored for specific wavelengths and integrating them into advanced photothermal systems. Additionally, further studies will explore the impact of nanoparticle coating materials and surrounding media on heat generation efficiency, aiming to extend the application of optimized AuNRs in biomedical and sustainable energy technologies.

## Data Availability

All data generated or analyzed during this study are included in this published article. The datasets used to create the figures are available from the corresponding author upon reasonable request.
